# Genetic Imprint of Vaccination on Simian/Human Immunodeficiency Virus Type 1 Transmitted Viral Genomes in Rhesus Macaques

**DOI:** 10.1371/journal.pone.0070814

**Published:** 2013-08-14

**Authors:** Mariana Varela, Ernst Verschoor, Rachel P. J. Lai, Joseph Hughes, Petra Mooj, Trevelyan J. McKinley, Timothy J. Fitzmaurice, Lisa Landskron, Brian J. Willett, Simon D. W. Frost, Willy M. Bogers, Jonathan L. Heeney

**Affiliations:** 1 Department of Veterinary Medicine, University of Cambridge, Cambridge, United Kingdom; 2 Department of Virology, BPRC, Rijswijk, The Netherlands; 3 MRC-University of Glasgow Centre for Virus Research, Institute of Infection, Immunity and Inflammation, College of Medical, Veterinary and Life Sciences, Glasgow, Scotland; Boston College, United States of America

## Abstract

Understanding the genetic, antigenic and structural changes that occur during HIV-1 infection in response to pre-existing immunity will facilitate current efforts to develop an HIV-1 vaccine. Much is known about HIV-1 variation at the population level but little with regard to specific changes occurring in the envelope glycoprotein within a host in response to immune pressure elicited by antibodies. The aim of this study was to track and map specific early genetic changes occurring in the viral envelope gene following vaccination using a highly controlled viral challenge setting in the SHIV macaque model. We generated 449 full-length env sequences from vaccinees, and 63 from the virus inoculum. Analysis revealed a different pattern in the distribution and frequency of mutations in the regions of the envelope gene targeted by the vaccine as well as different patterns of diversification between animals in the naïve control group and vaccinees. Given the high stringency of the model it is remarkable that we were able to identify genetic changes associated with the vaccination. This work provides insight into the characterization of breakthrough viral populations in less than fully efficacious vaccines and illustrates the value of HIV-1 Env SHIV challenge model in macaques to unravel the mechanisms driving HIV-1 envelope genetic diversity in the presence of vaccine induced-responses.

## Introduction

The development of a vaccine against human immunodeficiency virus type 1 (HIV-1) is a global health priority and is currently one of the greatest scientific challenges given the propensity of this virus to rapidly evolve within and between hosts. The phase III RV144 clinical trial in Thailand [1] generated a number of interesting leads regarding the immune correlates of protection, especially with respect to immune responses focussed on the HIV-1 envelope [2], [3]. The most intriguing finding from the RV144 trial is the correlation of protective efficacy of vaccine antibodies directed at the V1-V2 region of envelope. A major focus of prophylactic HIV-1 vaccines is the identification of envelope structures capable of inducing broadly neutralizing antibodies (NAbs). While the passive administration of neutralizing monoclonal antibodies (MAbs) alone have demonstrated convincing protection against a variety of viral challenges in pre-clinical models [4]–[10], the induction of broadly NAbs by immunisation with current recombinant forms of the HIV envelope glycoprotein (Env) remains elusive mainly due to the great variability of Env. While the discovery of broadly neutralizing MAbs and the detection of broadly neutralizing polyclonal sera from HIV-1 infected individuals provides evidence that this goal is achievable [11], [12], evidence supporting the role of non-neutralising anti-Env antibodies in vaccine-induced protection from infection has been growing.

Antibodies directed against Env have been shown to shape within-host virus evolution, to induce viral escape mutations [13] and are associated with slow disease progression in long-term non-progressors [14]. Only a few studies have taken the painstaking effort of thoroughly dissecting the immunological pressures and the molecular events of the autologous neutralising response in a small population of well-defined individuals infected with related variants [15]–[19]. In particular, the definition of epitopes that drive early neutralizing activity in response to Env vaccination has been greatly overlooked [20]. This has been deemed critical to the identification of regions that the virus cannot change without a great fitness cost considering there is increasing evidence demonstrating that there are limits to the extent of variation that the virus can tolerate [21]–[23]. This in turn has a direct impact on the development of novel vaccination strategies and antigens since traditional vaccination approaches have failed to induce broadly and potent NAbs against HIV-1. Sieve analysis comparing breakthrough viral populations between vaccine and placebo recipients is an important approach for evaluating the impact of putative immune correlates of protection [17], [19]. However, the complexity of the clinical setting in which the genetic composition of the viral population to which different individuals are exposed to, exact time of exposure, the dose, the different routes of infection and potential secondary exposures are compound variables that make the analysis of the vaccine immune response on different viral populations between hosts extremely difficult. Well-controlled pre-clinical vaccine studies in non-human primates however provide a unique opportunity to address these issues. The design of chimeric simian/human immunodeficiency virus (SHIVs) bearing HIV-1 *env* genes for pre-clinical vaccine evaluation allows a direct comparison between changes occurring in the *env* gene at the molecular level in a native context in the face of antibody responses. Despite the drawbacks such as the small groups of animals and the short duration of viremia with most virus challenge stocks, the use of HIV-1 *env* chimeric SIVmac (SHIV) viruses capable of infecting macaques has proven to be a strategically important model to assess HIV-1 Env vaccine candidates for efficacy *in vivo* prior to large-scale clinical trials.

In this study we aimed at identifying early specific changes occurring in the HIV-1 *env* gene under the pressure of pre-existing vaccine-induced immune responses and evaluate if sieve analysis could be used as a correlate of protection in SHIV pre-clinical efficacy studies. To this end, *env* genes were amplified directly from plasma using single genome amplification (SGA) from rhesus macaques that were part of a B-cell based pre-clinical vaccine trial and were challenged via the intrarectal route with SHIV_SF162P4_. Sequences derived from vaccinees and control animals were analysed and compared to search for signature changes associated with vaccine-induced antibody responses. This study provides a unique opportunity to examine the effects of pre-existing immunity on the evolution of the *env* gene without additional vaccine antigens as confounding factors since only Env-based antigens were used for immunization.

## Materials and Methods

### Ethics statement

The study involved a retrospective analysis of samples from an immunogenicity trial involving 20 adult rhesus macaques, weighing between 4 and 9 kg body and housed at the Biomedical Primate Research Centre (BPRC), The Netherlands. The trial included challenge with SHIV_SF162P4_. The trial protocol was approved (permit number DEC#515) by the Committee of the Ethics of Animal Experiments of Biomedical Primate Research Centre, Animal Welfare Assurance Number VVP/V 9513. The qualification of the members of this committee including their independence from a research institute is requested in the Wet op de Dierproeven (1996). The project was monitored by a qualified independent veterinarian, specially regarding the ethical issues of the project.

The use of non-human primates in The Netherlands is legalized based on the law: “Wet op de Dierproeven” and adaptations as published in the Staatscourant (48 (1975); 336 (1985); 585 (1992); 435 (1993); 806 (1994); 137(1996); 138(1996); 139 (1996); 5 (1997) and the EU guidelines 86/609/EEG. These laws guarantee the qualification of researchers, veterinary staff and animal caretakers involved in experimental studies and breeding of non-human primates. All animals were either from the breeding stock of the BPRC or purchased from breeding centers in Asia. Identification of imported macaques was confirmed by CITES. The accommodation of laboratory animals was in accordance with animal welfare requirements (1993); Wet op de dierproeven (WOD 1996); Gezondheids-en welzijnswet (GWWD 1996). The animal facilities were licensed to perform studies with genetically modified organisms up to DM3 level (Law on Genetically Modified Organisms, GMO law nr 108, 1996).

All steps were taken to ameliorate the welfare and to avoid the suffering of the animals. At the start of the trial, all animals were in good health and met with the following criteria: no previous immunosuppresive treatment; negative for simian T-lymphotropic virus, simian retrovirus and simian immunodeficiency virus (SIV); low or no IFN-γ, IL2 or IL4 responses against HIV Env, Gag, Pol or Nef antigens. They were housed in adjoining, single primate cages, because of the risk of cross-infection following challenge with SHIV. Animals could interact socially with their neighbors and had auditory and visual contact with others in the same room. Enrichment was provided in the form of pieces of wood, mirrors, food puzzles, variety of food and other home made or commercially available enrichment products. The facility was under controlled conditions of humidity (60%), temperature (23–25°C) and lighting (12 hour light/dark cycles). Animals were fed with standard food pellets, fruit and bread. Water was provided *ad libitum*. Animals were sedated with ketamin before blood was taken and challenge performed. The number of monkeys to be used in the trial was reduced to a minimum by statistical power calculations and variance values from previous studies to calculate the minimal group sizes to give statistical significance. All the included in this study were humanely euthanized at the end of the study, which was considered the human endpoint. Before euthanasia animals were sedated with ketamine and then received a lethal intravenous injection of pentobarbital.

At the BPRC all animal handling is performed in the Department of Animal Science (ASD) according to the laws as described above. At the BPRC a large experienced staff is available including full time veterinarians and pathologists. The ASD is regularly inspected by the responsible authorities (VWA) and an independent Animal Welfare Officer.

All animals were Mamu A*001, B*008 and B*017 negative except BB204 (A*001 postive), R01070, R00042, BB204, 98041, 96050 (B*008 positive) and R01012 (B*017 positive).

### Study design

Twenty Indian rhesus macaques (*Macaca mulatta*) were randomized into four groups of five and included in the study. Animals were primed at week 0 and boosted at weeks 6 and 16 as follows: Group 1 (gp140 glycoproteins): three injections with recombinant o-gp140 from HIV-1 Q461 (subtype A), HIV-1 SF162 (subtype B) and HIV-1 TV1 (subtype C); Group 2: week 0, one injection with HIV-1 SF162 V2 peptide, HIV-1 SF162 V3 cyclised peptide and CD4 binding site mimotope; week 6 same as week 0 with the addition of the gp140 glycoproteins used in Group 1; and week 16 gp140 proteins alone; Group 3: week 0 one injection with a membrane proximal external region peptide, a second CD4 binding site mimotope (different from the one administered to Group 2) and a HIV-1 TV1 V3 cyclised peptide; week 6, the same immunogens plus the same gp140 glycoproteins used in Group 1; and only the gp140 glycoproteins at week 16. Group 4: three injections with adjuvant alone. All animals were challenged intra-rectally at week 24 with 1,800 TCID_50_ of the cell-free challenge stock SHIV_SF162P4_ [24], [25].

### Immunogens

Gp140 glycoproteins (50 μg), cyclised V3 peptides (250 μg) or linear peptides (250 μg) conjugated to keyhole limpet haemocyanin (180 μg) were formulated in MF59 adjuvant plus CpG. o-gp140s have five amino acid mutations in the SU-TM cleavage site. Proteins were purified from CHO supernatant using a combination of GNA, DEAE and CHAP columns. At the final stage, trimers are separated from the monomers using gel filtration column. The sequences of the synthetic peptides used were the following: HIV-1 SF162 V3 CTRPNNNTRKSITIGPGRAFYATGDIIGDIRQAHC; HIV-1 TV1 V3 CTRPNNNTRKSVRIGPGQAFYATNDVIGNIRQAHC; HIV-1 SF162 V2 IRNKMQKEYALFYKL; HIV-1 TV1 V2 LRDKKHKEYALFYKL; mimotope to monoclonal IgG1 b12: NWPRWWEEFVDKHSS [26] (Group 2); mimotope to monoclonal IgG1 b12: HERSYMFSDLENRC [27] (Group 3); Membrane proximal region NEQEL LELDK WASLWN [28]. A C-terminal cysteine was added to all peptides for conjugation to keyhole limpet haemocyanin.

### Viral load determination

Plasma virus load was determined by a quantitative competitive reverse transcription-PCR as previously described [29].

### Neutralization assays

Neutralizing titers of sera were assessed using single-round competent viruses bearing an Env protein of the SHIV_sf162p4_ virus inoculum or specific Env identified by the evolutionary analysis and TZM-bl cells as a target as previously described [30]. Briefly, virus was incubated with serially diluted antisera for 1 hour at 37°C before being added to wells of 96-well microplates seeded with TZM-bl cells. After 48 hours cells were lysed and luciferase signal in the lysate was developed with Britelite Plus substrate (Perkin Elmer) and read on a VICTOR Light luminometer (Perkin Elmer).

### ELISAs

Binding antibodies were determined in plasma or serum by ELISA. In brief, ELISA plates were coated with the indicated antigens and plates were blocked with PBS containing 0.1% Tween20, 4% BSA and 1% newborn calf serum. Subsequently, plates were incubated with EDTA plasma or serum samples diluted in blocking buffer. After washing in a PBS/0.1% Tween20 buffer the wells were incubated with an alkaline phosphatase (AP)-conjugated goat anti-human IgG antibody (1∶1000, Sigma). pNPP substrate (Sigma) in Tris buffer was used for colour development. The reaction was stopped by the addition of H_2_SO_4_ and colour intensity was measured on a BIORAD microplate reader at 405 nm (Biorad, model 680).

### ELISpots

The quantification of antigen-specific cytokine-secreting cells was performed as previously described by Koopman et al. [31]. The various antigens were used to measure antigen-specific immune responses, medium alone was used as negative control, whilst phorbol myristate acetate (PMA) (20 ng/ml) plus ionomycin (1 μg/ml) stimulation was used as positive control. In brief, 4×10^6^ cells/ml were stimulated for 24 hours. For the enumeration of antigen specific cytokine production, non-adherent cells were collected and plated at 2×10^5^ cells/well in triplicate in a 96-well ELISpot plate with the same antigen again. The microtiter plates were pre-coated with MAbs specific for the lymphokines anti-IFN-γ MAb (MD-1, U-Cytech, Utrecth, The Netherlands), anti-IL-4 MAb (QS-4, U-Cytech) and anti-IL-2 MAb (B-G5, Diaclone Laboratories, Besançon Cedex, France). Detection of the cytokine secreting cells took place after 15 hours for IL-4 and 4 hours for IFN-γ as well as IL-2. The cells were lysed and the debris was washed away before adding detector antibodies. IFN-γ, IL-2 and IL-4 were detected using biotinylated rabbit-anti-rhesus macaque IL-2, biotinylated rabbit-anti-rhesus macaque IFN-γ, or biotinylated mouse-anti-rhesus macaque IL4 (U-Cytech). Spots were visualized using streptavidin-HRP and an AEC (3-amino-9-ethylcarbazole) colour development system. Two background responses (medium only) were subtracted.

### Viral RNA extraction and cDNA synthesis

Viral RNAs were extracted from 200 µl using a QIAamp viral RNA kit (Qiagen) following the manufacturer's instructions. Viral RNA was reverse-transcribed using Superscript III (Invitrogen) as previously described [32].

### Single genome amplification

Nested PCRs were performed using a cDNA dilution that yielded less than 30% positive reactions in a 96 well plate at which the majority of positive reactions would result from the amplification of one single copy of cDNA. PCRs and nested PCRs were performed using Platinum Taq DNA Polymerase High Fidelity^®^ (Invitrogen) following manufacture's instructions. BFwout 5′GCAATAGTTGTGTGGTCCATAGTAATCATAG3′ and SHIVR2 5′GCCTCACTGATACCCCTACC3′ primers were used for first round PCRs and BFW162-AC AATAGACCGGTTAATCGATAGAATAACAG and SHIVp4RW 5′TCCTCTAGACCCTGATTGTATTTCTGTCC3′ primers were used for second round PCRs. 5 μl of the PCR product was treated with ExoSap-IT^®^(USB) diluted and sequenced directly using an Applied Biosystems 3730 DNA Analyzer. Partially overlapping primers were used and both DNA strands were sequenced. Amplification from a single template was verified by the inspection of individual chromatograms and sequences with mixed based were excluded from the analysis.

Samples from animals R01093 week two, R00057 week four and BB204 week 4 post-challenge were subjected to bulked PCR following the same protocol and conditions as for the SGA samples. These three PCR products were then cloned using TOPO XL PCR cloning kit (Invitrogen) following manufacture's instructions except that MAX Efficiency® Stbl2 (Invitrogen) chemically competent cells were used for transformation. Individual bacterial clones were picked up, grown for 24 hours at 30°C when plasmid DNA was extracted. Sequencing was performed as described above.

### Evolutionary analysis

Individual *env* sequence reads were assembled using the Lasergene SeqMan (DNASTAR) package. Sequences were manually aligned using Se-Alv2.0a11 Carbon (http://tree.bio.ed.ac.uk/software/seal/). Protein sequence alignments were performed using MAFFT-6.859 (http://mafft.cbrc.jp/alignment/software/). All sequences were tested for hypermutation by APOBEC3G/F with Hypermut 2.0 (www.hiv.lanl.gov). Recombination was screened by the Genetic Algorithms for Recombination Detection (GARD) [33] available in the Datamonkey web interface of the HyPhy software package using the TrN93 nucleotide substitution bias model and beta-gamma site-to-site variation (2 rate classes).

Maximum likelihood (ML) trees were estimated using PhyMl 3.0 [34] and RAxML 7.2.8 [35], [36] using the generalized time reversible substitution model. Sequence diversity was estimated using MEGA 5 [37] after the removal of hypermutated sequences. The mean protein sequence divergence from a reference (vaccine sequence) was estimated using DIVEIN based on phylogenetic trees rather than pairwise comparisons [38] and differences among groups were compared using ANOVA followed by Tukey's to identify groups. Mean number of non-synonymus (dN) and synonymus (dS) substitutions per site (ratio dN/dS) were estimated using the Single Likelihood Ancestor Counting (SLAC) algorithm available in the Datamonkey web interface of the HyPhy software package [39] using alignments stripped of hypermutated sequences. To address the number of potential N- linked glycosylation sites nucleotide sequences were translated into amino acid sequences using Se-Alv2.0a11 Carbon and then were screened by N-GlycoSite [40]. Shannon entropy was calculated using Entropy-2 (www.hiv.lanl.gov).

All statistical calculations were carried out using R 2.14.1 [41] or GraphPad Prism.

### Accession numbers

All sequences were deposited in GenBank (accession numbers JX191370 to JX191881).

### Online supplemental material

A summary of the sequences analysed is shown in Table S1.

## Results

### Vaccine trial outcome

**Figure 1:**
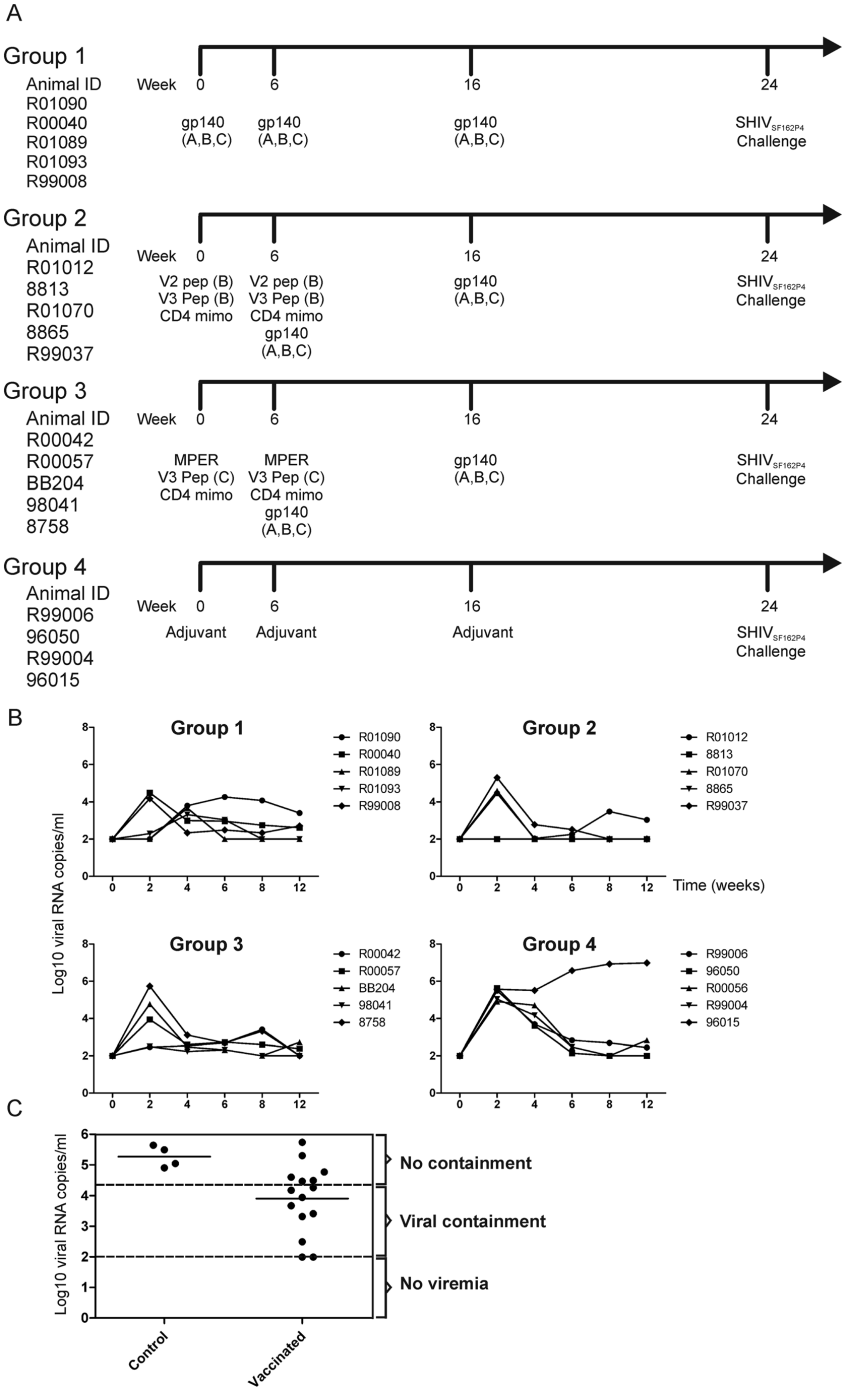
Trial outcome. **A) Schematic representation of the experimental design. B) Infection kinetics after mucosal challenge with SHIV_SF162P4_.** Viral loads were measured at the indicated times post infection for a period of 12 weeks in each of the experimental groups. Since the detection level of the assay was 100 viral copies/ml negative samples were assigned a value of 2 (Log10 of 100). **C) Distribution of peak viral RNA loads.** Continuous lines indicate the means of each group; upper discontinuous line indicates viremia 1 log lower than the mean peak viremia of control animals while lower line indicates the limit of detection of the assay (100 viral RNA copies). Control animal 96015 was not included in the graphic and calculations since its viral load continue to increase during the study period.

The development of an HIV-1 vaccine has been elusive. While impressive protection from SHIV challenge has been achieved by passive immunisation with neutralising monoclonal antibodies *in vivo*, recapitulation of these impressive results by active vaccination has not been achieved with current trimeric forms of the HIV-1 envelope. One likely explanation is that the polyclonal antibody response to the global gp140 immunogen insufficiently amplifies antibodies to the relatively few but important neutralising epitopes clustered at certain sites of the structure. Thus, we designed a study in which animals would first be immunised with peptides corresponding to neutralising epitopes (ie, MPER, V3, V2, CB4bs) to prime a B-cell response to these epitopes, and then amplify those by boosting with a conformationally correct gp140 (gp140 trimer). To this end, twenty Indian rhesus macaques were divided into three groups of five animals (groups 1 to 3) that were immunized and one group of five (group 4) that was used as an adjuvant alone control. Immunized animals received a combination of gp140 envelope proteins from subtypes A, B and C and peptides corresponding to V2, V3, CD4 binding site and membrane proximal external region (MPER) as described in methods and shown in Figure 1A. Eight weeks after the last immunization all animals were exposed to a single dose of 1,800 TCID_50_ of SHIV_SF162P4_ virus stock via the intrarectal route [42].

As a measure of protection, viral loads were monitored from the day of challenge and at regular intervals up to twelve weeks post-challenge (Figure 1B). We evaluated protection from infection by comparing: i) viral loads at week 2 post challenge; ii) the 8-week area under the curve; and iii) peak viral loads. For viral loads at week 2, we found statistically significant differences between groups, in particular between control animals and animals from group 1 (Kruskal-Wallis test, p<0.05; Dunn's multiple comparison test, group 1 vs group 4: p<0.05). For the 8-week area under the curve, we found statistically significant differences between groups, in particular between control animals and animals from group 2 (Kruskal-Wallis test, p<0.05; Dunn's multiple comparison test, group 2 vs group 4: p<0.05). For peak viral loads, we did not find statistically significant differences between controls and vaccinees, however a delay in peak viral loads was detected in some animals belonging to group 1.

Moreover, we found that 4 out of 5 animals of group 1 had peak viral loads that were at least one log lower than the mean peak viral load of control animals. Indeed, one of these animals had just a transient detectable sample and remained aviremic during the length of the study. Interestingly 2 animals of group 2 were aviremic throughout the study while another animal had a detectable transient viremia at week 2 post-challenge, although the later was positive for the MHC class I allele Mamu B*008, which is associated with control of SIVmac239 infection. Finally, 3 animals of group 3 had peak viral loads that were at least one log lower than the mean peak viral load of control animals.

In summary, if we consider a reduction in viral load of at least one log with respect to control animals a measure of partial viral containment as previously utilized by others [43], [44], then 2 animals were completely protected from infection, another 2 had transient infections and a further 7 animals were partially protected by this vaccine strategy, providing a combined 66% complete and partial protection (Figure 1C).

### Pre-challenge immune responses

**Figure 2:**
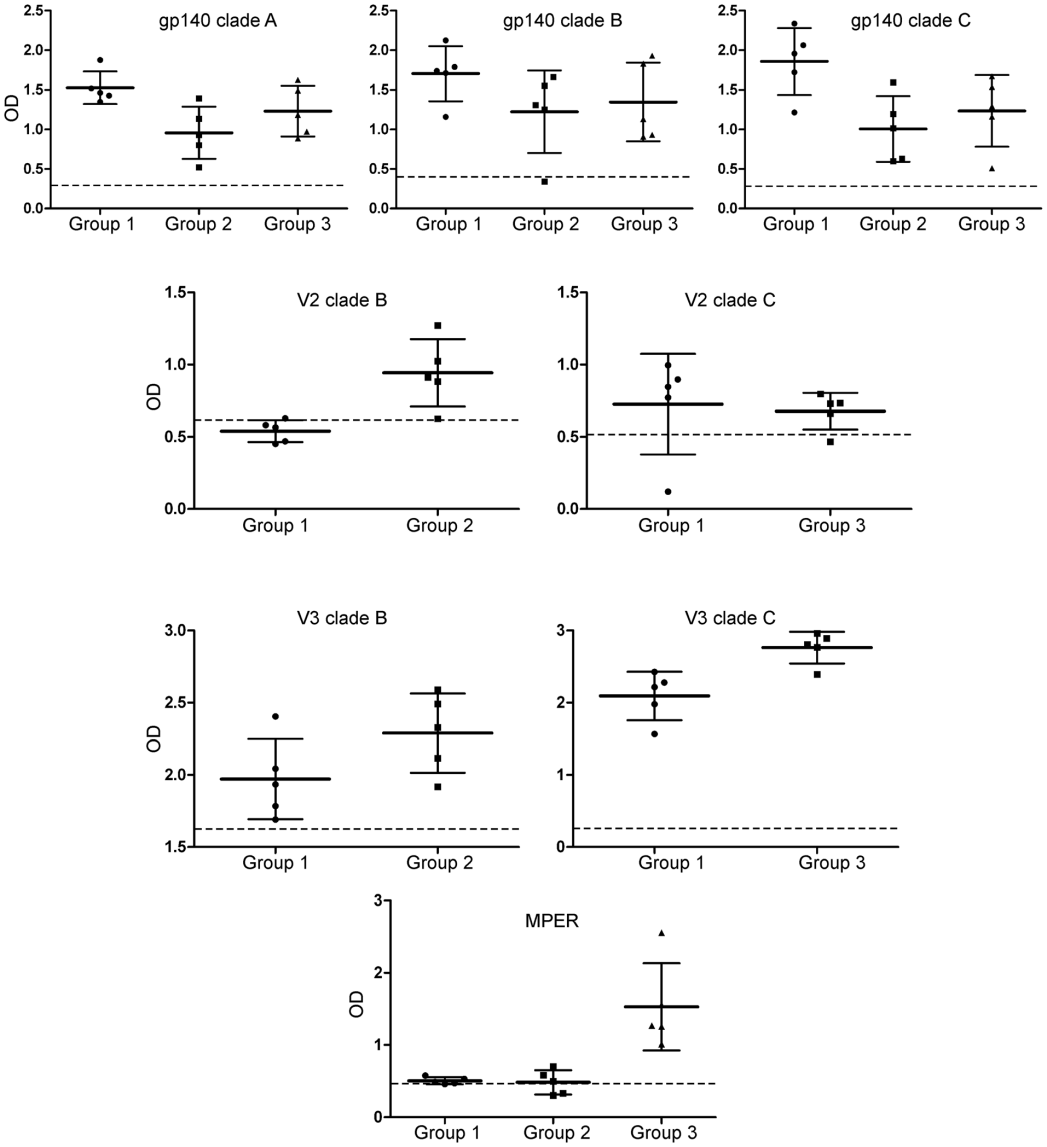
Binding antibodies after the last immunization. The presence of binding antibodies against gp140 or the indicated peptides used for immunization was measured using ELISA at a single serum dilution. The sera collected after 2 weeks of the last immunization was analysed against gp140 while the sera collected 6 weeks the last immunization was analysed against the indicated peptides. The interrupted lines depict the threshold OD value above which a sample is considered positive (3 times the OD value of the pre-immunization sera).

Antibody (Ab) titers were monitored along the course of immunization. All vaccinated animals developed Env-specific Abs against the three subtypes after the third immunization as determined by ELISA using recombinant gp140 (Figure 2). In addition, animals from groups 2 and 3 that were primed with peptides against V2, V3 and MPER developed binding antibodies against those regions (Figure 2). In some cases, animals from group 1 also developed antibodies against those regions. Most importantly, all animals belonging to the vaccinated groups developed NAbs against SHIV_SF162P4_ measured by the TZM-bl assay before challenge while no neutralizing activity was detected in the serum of control animals (Table 1). In general, we found that the animals that had the highest peak viral loads were the ones that had the lowest NAb titres, a trend that was confirmed for all the vaccinated groups, although this correlation only reach statistical significance for animals of group 2 (Pearson correlation coefficient, -.088; P<0.05).

**Table 1:** Pre-challenge neutralizing antibody responses.

	Pre-immune	Week 14	Week 22	Week 24	Week 36
Group 1					
R01090	<20	33	2018	909	18498
R00040	<20	<20	1345	474	3538
R01089	<20	39	5001	1624	6687
R01093	<20	<20	3775	752	24800
R99008	<20	<20	891	485	7830
Group 2					
R01012	<20	<20	550	360	>87480
8813	<20	<20	969	602	160
R01070	<20	<20	566	441	2762
8865	<20	<20	1896	669	272
R99037	<20	<20	116	24	785
Group 3					
R00042	<20	<20	860	511	>15884
R00057	<20	<20	1081	952	>15887
BB204	<20	<20	781	800	16998
98041	<20	<20	855	474	81
8758	<20	<20	433	261	7063
Group 4					
R99006	<20	<20	<20	<20	3680
96050	<20	<20	<20	<20	19781
R00056	<20	<20	<20	<20	41620
R99004	<20	<20	<20	<20	42643
96015	<20	<20	<20	<20	<20

Sera collected at different time points before and after challenge were tested against single-round competent virus bearing an Env protein of the SHIV_sf162p4_ virus inoculum. The reciprocal of serum dilutions at which 50% inhibition of viral infection (IC_50_) occurred are reported.

**Figure 3:**
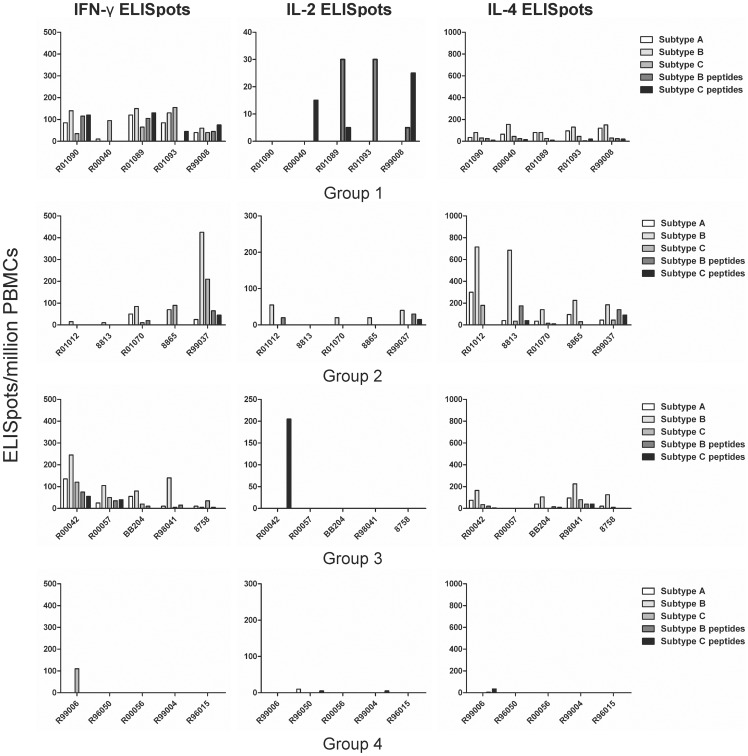
T-cell ELISpot responses 2 weeks after the last immunization. Positive IFN-γ, IL-2 and IL-4 ELISPots per million cells are shown for each animal after stimulation with full-length recombinant proteins of clades A, B and C and V2-V3 peptides of clades B and C once 2 background responses were subtracted. Positive responses are defined as responses above 50 spot forming units per million cells.

The presence of cellular immune responses was evaluated by quantification of antigen-specific cytokine-secreting cells (IFN-γ, IL-2 and IL-4) by enzyme-linked immunospots (ELISpot) on fresh PBMC isolated 2 weeks after the last immunization (Figure 3). Most of vaccinees had IFN-γ-secreting cells after stimulation with recombinant proteins of HIV clades A, B and C while no responses were detected in naïve control animals. We found no correlation between peak viral loads and the development of cellular responses.

### Vaccination does not constrain the transmission bottleneck

**Figure 4:**
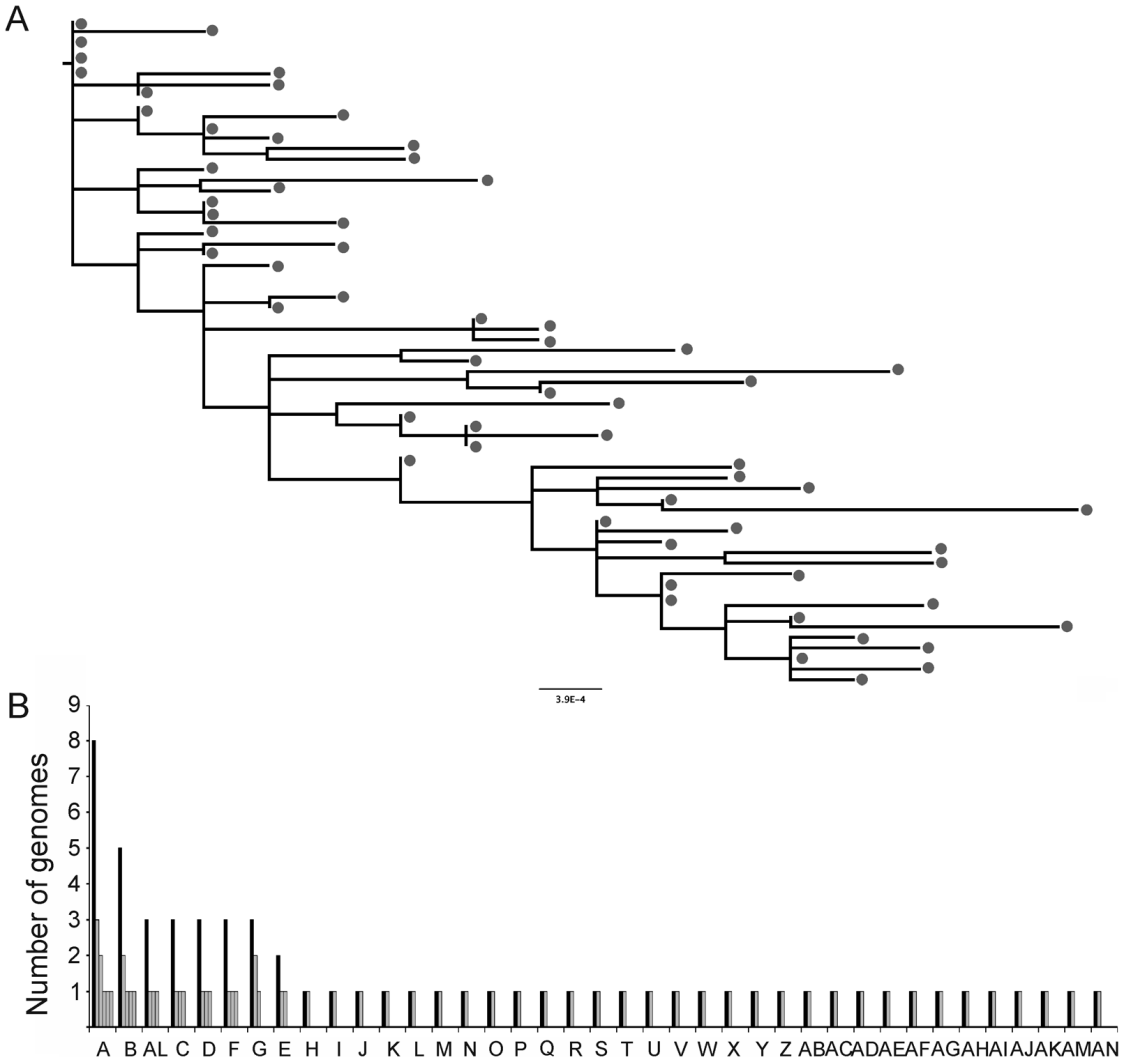
A) Maximum likelihood phylogenetic tree of the envelope gene of the virus inoculum. The scale bar represents one nucleotide substitution. **B) Frequency of each inoculum variant at the amino acid level.** The number of each viral genome present within the inoculum was estimated based on the amino acid sequence (black bars) and at the nucleotide level (grey bars).

To characterize the impact that pre-existing immunity had on the breakthrough viral population, full-length *env* sequences were derived from plasma samples taken 2 and 4 weeks post-challenge from 6 vaccinees and 2 control animals. As this was a retrospective study only of subset of unbiased samples were available for sequence analysis and unfortunately no samples were available from animals belonging to Group 2. However, we included sequences of two animals (Ri102 and Ri112) as extra controls that were part of a previous study [32] and that were challenged with the same inoculum and route and had comparable viral loads and kinetics of infection to the contemporary controls. 512 sequences, including 63 sequences from the virus inoculum, were obtained using SGA followed by direct sequencing (19 to 60 per animal; median 46) (table S1). This virus inoculum has been previously characterized [32], however additional genomes were included for this analysis. The phylogenetic relationships among sequences were estimated using maximum likelihood (Figure 4A). The inoculum was characterized by the lack of a majority variant. In fact, only 8 out of the 63 inoculum variants were present at a number higher than one. Randomly distributed and shared polymorphisms were identified. When nucleotide sequences were translated to amino acids, 38 unique phenotypes were identified. The number of each of these unique phenotypes is shown in Figure 4B.

**Figure 5:**
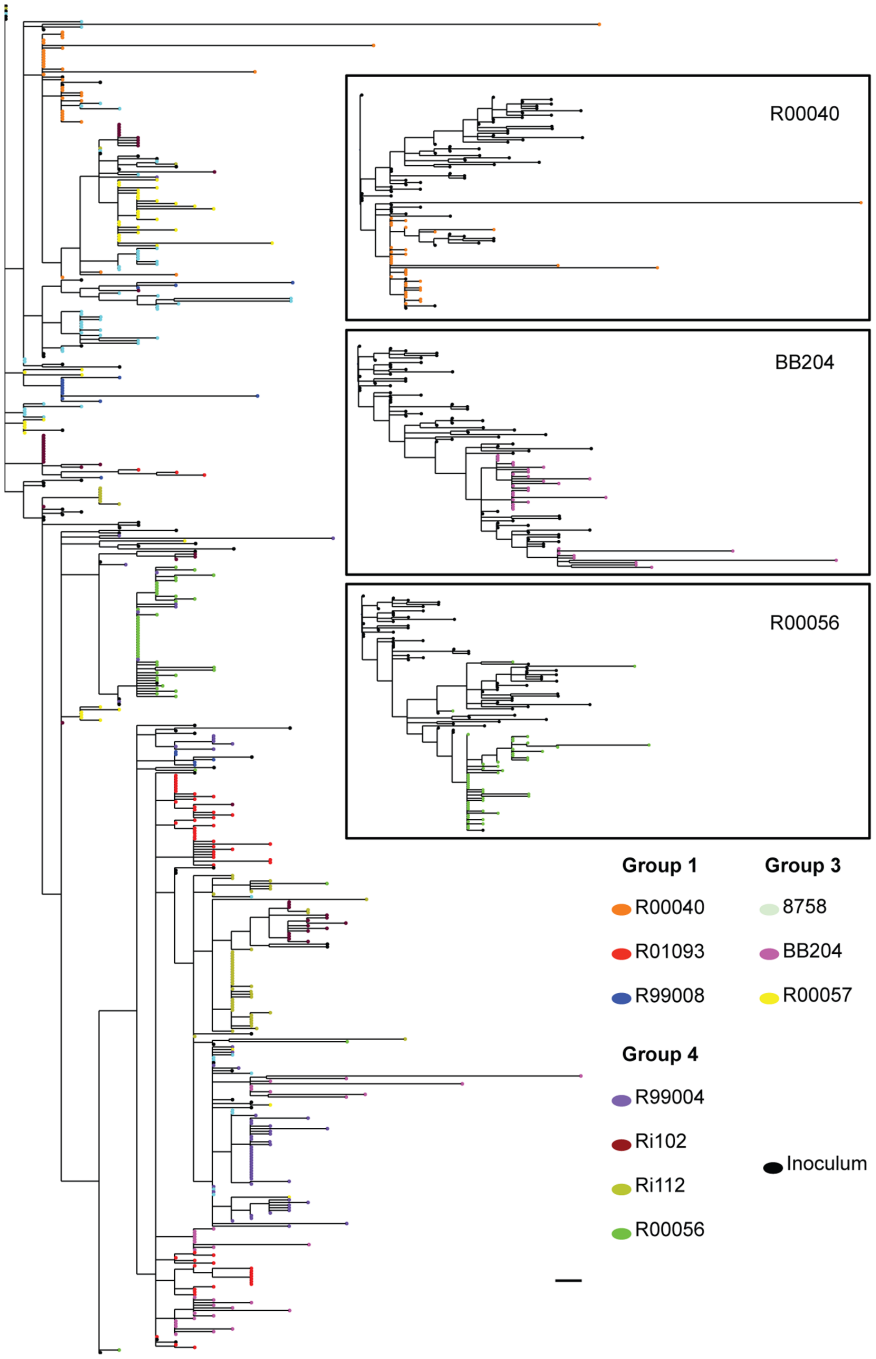
Composite phylogenetic tree of all envelope sequences along with sequences of the challenge inoculum. Master phylogeny shows no clustering according to vaccination status. Insert shows representative intrahost phylogenies. Animal-derived sequences are color-coded. Black circles indicate inoculum-derived sequences. The scale bar indicates one nucleotide substitution.

To estimate the number of viral genomes (founder viruses) that established infection upon challenge the phylogenies of each intrahost data set were reconstructed using maximum likelihood (not shown). Multiple transmission events/founder viruses were identified when lineages shared 2 or more mutations as defined by Keele et al. [45]. A phylogeny reconstructed using all the available sequences (Figure 5), including sequences from the virus inoculum, revealed no particular clustering according to vaccination status. Multiple viruses established infection in all of the animals, which was revealed by the complex interspersed sequences across the tree. This is in agreement with our previous study where the same virus inoculum, infection route and dose were used [32]. The total number of transmissions (founder viruses) for each animal is shown in Table 2. We found no statistically significant differences in the number of transmissions/founder viruses between controls and vaccinated animals, which is not surprising considering that: 1) the use of such a high inoculum dose made this a very stringent challenge model; and 2) the immunization protocol had little clinical benefit in terms of protection from infection.

**Table 2:** Number of founder viral variants.

	Group 1			Group 3			Group 4 (controls)			
	R00040	R01093	R99008	R00057	BB204	8758	R00056	R99004	Ri102	Ri112
Number of founder viruses	4	5	3	6	3	11	6	6	6	7

### Global filter analysis

**Figure 6:**
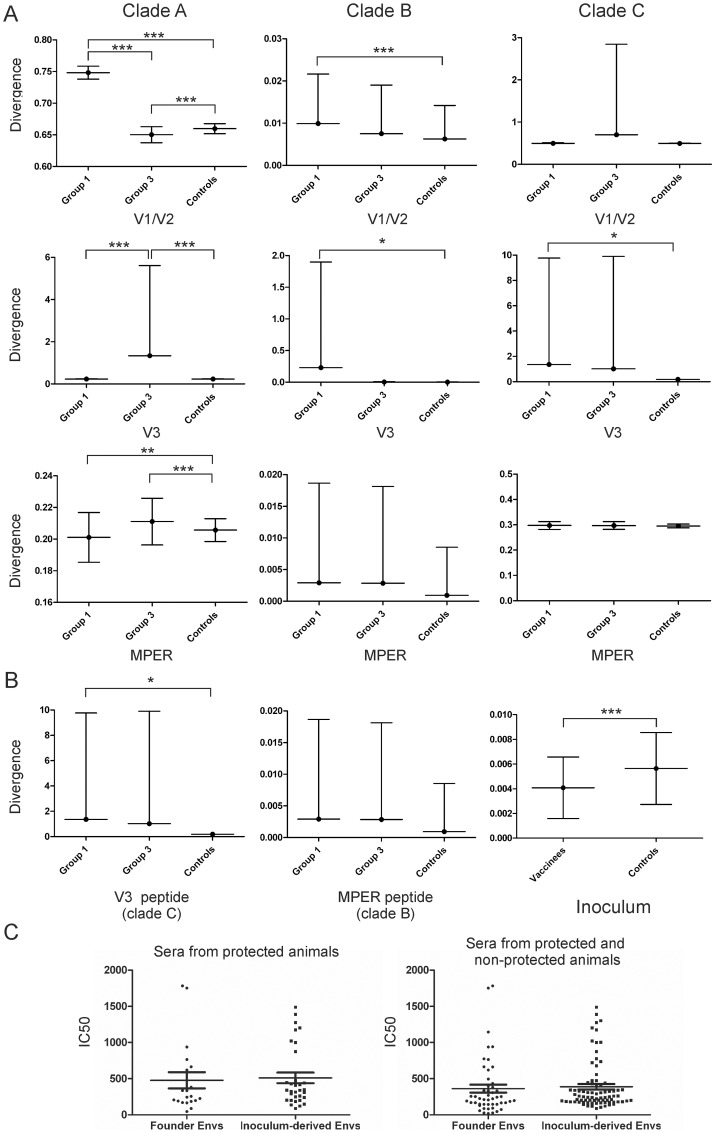
Protein divergence between animal sequences and vaccine inserts. The mean protein divergence from the protein sequences included in the vaccine regime (gp140 in A and peptides used in boosting in B) and standard deviation are reported. Only statistically significant comparisons are indicated. C) The reciprocal of serum dilutions at which 50% inhibition of viral infection (IC_50_) occurred from sera derived from the day of challenge from animals that were protected (left) or protected and non-protected (right) against pseudotypes harbouring Envs derived from the virus inoculum or animal plasma (founder Envs) were compared. No statistically significant differences were identified (un-paired t-test).

Firstly, we estimated the mean protein sequence divergence from the vaccine inserts and compared it among groups (ANOVA followed by Tukey's test as a post hoc test). We found a statistically significant difference between controls and vaccinees in the V1/V2 region when the divergence was calculated against clade A and B vaccine inserts (Figure 6A, top panels). For the V3 region we found statistically significant differences in divergence against the 3 clade vaccine inserts between vaccinees and controls (Figure 6A, middle panel). When the divergence was estimated and compared for the MPER we only found statistically significant differences from the clade A gp140 vaccine insert (Figure 6A, bottom panel). In the majority of the cases where we found significant differences between controls and vaccinees, the divergence was higher in vaccinees. When the divergence from the peptides used for priming group 3 was compared among groups (Figure 6B) we found that it was higher in groups 1 and 3 compared to control although this difference did not reach statistical significance. The divergence from the CD4 binding site mimotope was not calculated because this peptide was shown not to be immunogenic [46]. On the other hand, we observed that the divergence from the consensus sequence of the inoculum was greater in controls than in vaccinees (t-test p<0.01) (Figure 6B, right panel). In fact, we cloned 8 *envs* derived from the inoculum and 5 envs derived from animals (founder *envs*) and tested their sensitivity to neutralization against the day of challenge sera from animals that were considered protected (R8813, R8865, R01089 and R01070) versus the sera of protected and non-protected animals (R8813, R8865, R01089, R01070, R00040, R01093, R99008, R00057, BB204 and 8758) and we found no differences in neutralization which indicates that Envs in the inoculum and founder Envs are similar in terms of neutralization sensitivity (Figure 6C).

We found that vaccinated animals had a higher proportion of sequences harbouring deletions (vaccinated median  = 0.2 versus control media  = 0.055; Wilcoxon test, p<0.05), indicating that there was a higher tendency for the generation of aberrant genomes in vaccinated animals. Most of the deletions were not in-frame and there were no compensatory mutations to maintain an open reading frame. Six genomes had large deletions of more than 100 amino acids in-frame, which likely give rise to non-functional proteins.

We did not find changes in the number or distribution of predicted sites for N-linked glycosylation between vaccinated and control animals suggesting that if NAbs impose pressure to drive early diversification events this does not occur primarily by changing glycosylation sites. This is in agreement with the findings of Wood et al. in acute subtype B infections in humans [47].

### Local filter analysis

**Figure 7:**
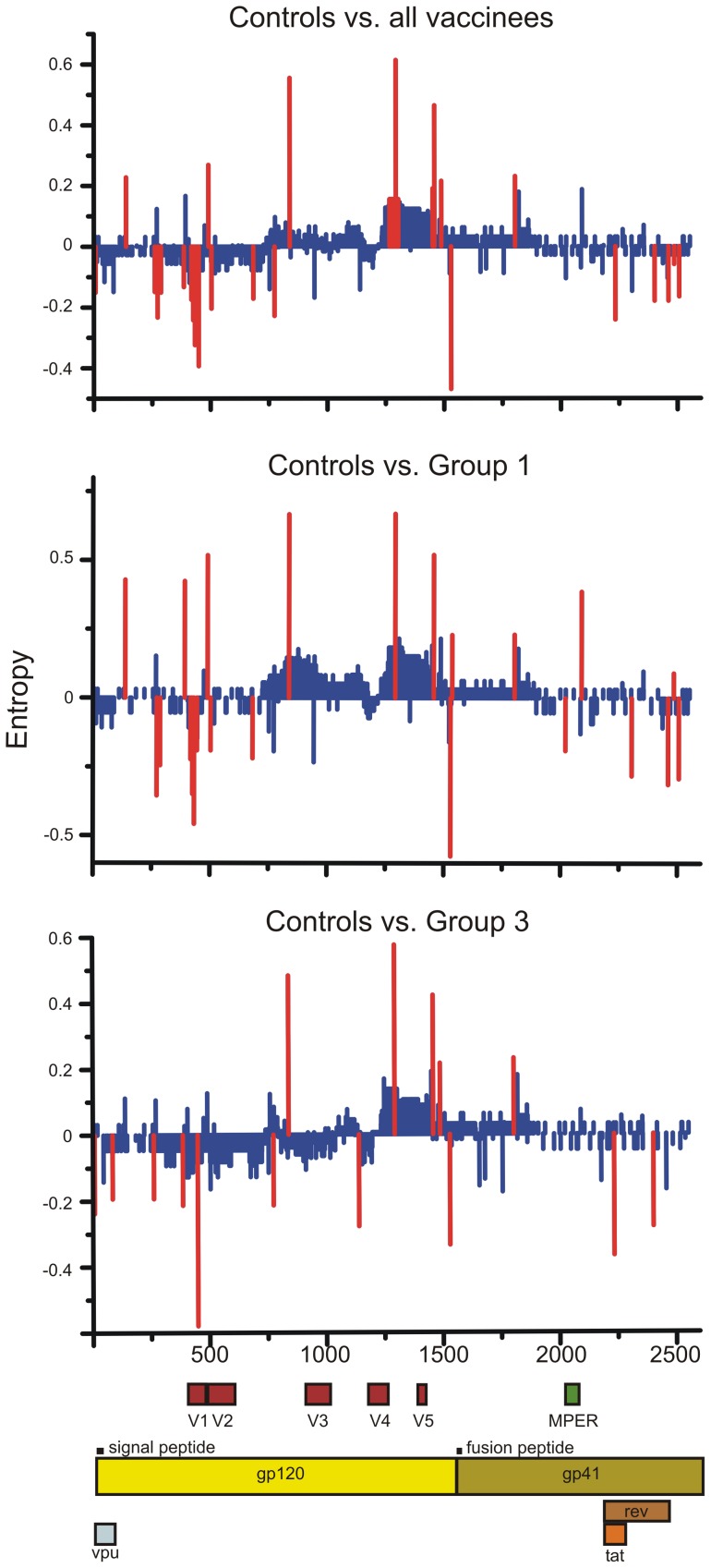
Differences in Shannon entropy between controls and vaccinees. The Shannon entropy was calculated at each nucleotide position for pooled vaccinees, group 1 vaccinees and group 3 vaccinees and subtracted from the entropy calculated for control animals. Sites displaying a statistically significant difference in entropy between controls and vaccinees are indicated in red.

We next sought to identify nucleotide sites that showed a significant difference in variability between controls and vaccinees. To this end the Shannon entropy was estimated at each nucleotide position and was compared between vaccinees and controls using a Monte Carlo randomization strategy implemented in Entropy-2. We identified a total of 74 nucleotide sites where there was a statistical significant difference in the Shannon entropy between control animals and vaccinees (Table 3 and Figure 7). In 25 of these sites the Shannon entropy and thus diversity was higher in vaccinees while in 49 sites the diversity was higher in control animals indicating that vaccinees were constrained in the number of sites in which diversification could occur (Fisher exact test *P*<0.05). The differences in Shannon entropy between controls and vaccinees seemed to occur in clusters, where several sites in close proximity displayed higher entropy for either controls or vaccinees and not as isolated single sites probably due to genetic linkage. In particular, we identified two major regions in which the Shannon entropy was higher in vaccinees: from amino acids 87 to 96 (HXB2 88 to 97) in C1 and from amino acids 140 to 150 (HXB2 141–152) in V1. In control animals the Shannon entropy was higher in two other regions: from amino acids 422 to 435 (HXB2 429–442) in C4 and 482 to 486 (HXB2 488–492) in C5. This suggests that different regions of the envelope underwent different patterns of diversification in vaccinees and control animals.

**Table 3:** Positions showing statistically significant differences in Shannon entropy between controls and vaccinees.

Site Nucl/aac	HXB2 site Nucl/aac	Location	All vaccinees vs controls (p<0.005)	Group 1 vs controls (p<0.005)	Group 3 vs controls (p<0.005)	Selection	Type of mutation	Bayesian model
7/3	7/3	Vpu/signal peptide	yes	no	yes	no	non syn	no
84/24	86/29	Signal peptide	no	no	yes	no	syn	no
138/46	140/47	C1	yes	yes	no	negative	syn	R99004
261/87	263/88	C1/glycosite 88	yes	no	yes	no	non syn	no
270/90	272/91	C1	no	no	no	no	syn	Ri112
273/91	275/92	C1	yes	yes	no	no	syn	no
288/96	290/97	C1	yes	yes	no	no	syn	no
386/129	388/130	C1	yes	no	yes	no	non syn	no
393/131	395/132	V1	no	yes	no	negative	syn	Ri112
406/136	408/136	V1	no	no	no	no	non syn	no
418/140	420/141	V1	yes	yes	no	no	non syn	no
422/141	424/142	V1	yes	no	no	no	Syn	no
426/142	429/143	V1	yes	yes	no	no	Non syn	no
433/145	435/146	V1	yes	yes	no	no	Non syn	no
445/149	450/151	V1	yes	yes	no	no	Non syn	no
449/150	454/152	V1	yes	no	yes	no	Non syn	R00057
491/164	496/166	V2	yes	yes	no	positive	Non syn	R00056
504/168	509/170	V2	yes	yes	no	no	Non syn	no
683/228	685/229	C2	yes	yes	no	no	Non syn	no
753/251	755/252	C2	no	no	no	negative	Syn	Ri102
759/253	761/254	C2	no	no	no	negative	Syn	R01093, R99004, Ri112
774/258	776/259	C2	yes	no	yes	no	Syn	no
839/280	841/281	C2	yes	yes	yes	no	Non syn	R00056
997/333	1002/334	C3	no	no	no	no	Non syn	R01093, R99004, BB204
1009/337	1014/338	C3	no	no	no	no	Non syn	R01093, R99004, BB204
1140/380	1145/382	C3	no	no	yes	no	Syn	no
1266–1304/422–435	1285–1324/429–442	C4	yes	no	no	no	N/A	no
1294/432	1314/439	C4	yes	yes	yes	no	Non syn	R00056
1314/438	1334/445	C4	no	no	no	negative	Syn	R01093, BB204
1445/482	1462/488	C5	yes	no	no	no	Syn	no
1446/482	1463/488	C5	yes	no	no	no	Syn	no
1451/484	1468/490	C5	yes	no	no	no	Non syn	no
1458/486	1475/492	C5	yes	yes	yes	negative	Syn	R00056
1488/496	1505/502	C5	yes	no	yes	no	Non syn	no
1530/510	1544/515	GP41/fusion peptide	yes	yes	yes	no	Syn	R00040
1537/513	1551/518	GP/41fusion peptide	no	yes	no	No	Non syn	R00057
1754/585	1767/589	GP41					Syn	R01093
1764/588	1778/593	GP41	no	no	no	No	Syn	Ri112, BB204
1804/602	1818/607	GP41	yes	yes	yes	No	Non syn	no
2022/674	2013/672	GP41/MPER	no	yes	no	No	Syn	no
2091/697	2102/701	Gp41	no	yes	no	negative	Syn	no
2234/745	2245/749	Gp41/rev/CT	yes	no	yes	No	Non syn	BB204
2305/769	2311/771	Gp41/rev/CT	no	yes	no	No	Non syn	R99008
2402/801	2413/805	Gp41/rev CT	yes	no	yes	No	Non syn	no
2461/821	2472/825	Gp41/CT	yes	yes	no	No	Non syn	R99008
2485/829	2496/833	Gp41/CT	yes	yes	no	No	Non syn	R01093, R00056
2507/836	2518/840	Gp41/CT	yes	yes	no	No	Non syn	no

The sequences of all vaccinees were compared against the sequences of all control animals or the sequences of vaccines were divided according to the vaccination protocol (groups 1 and 2) and compared against controls. The last column shows the nucleotide sites-of-interest identified using a Bayesian model.

CT: cytoplasmic tail.

no: sites not identified by the Bayesian tool.

Intriguingly, despite the presence of binding antibodies against V3 in both groups 1 and 3, as measured by ELISA (Figure 2), there were no sites showing significant differences in entropy between controls and vaccinees in this region. In contrast to the differences in Shannon entropy between controls and vaccinees that seemed to occur in clusters in gp120, all the sites identified in gp41 appeared isolated. Interestingly most of the identified sites were located in the cytoplasmic tail. This together with the identification of sites in the signal peptide suggest that the level of expression and incorporation of the envelope might be important to sustain viral replication in the presence of antibody pressure.

### Identification of nucleotide sites-of-interest

A usual concern that arises when performing the type of analysis described above is the identification of nucleotide sites that are not the result of simply random *de novo* mutation events occurring during viral replication or *in vitro* amplification. Thus, to identify nucleotide sites that showed strong evidence of change in the distribution of bases relative to the inoculum, we applied a Bayesian technique that has been used successfully for similar HIV-1 and flu intra-host data sets before [32], [48], [49]. A total of 21 nucleotide sites were recognized using this approach of which 11 were non-synonymous mutations (Table 3). Six sites were present in more than one animal, which could be indicative of sites that are important for transmission or early replication events. A single site (nucleotide 1314; HXB2 1334) showed evidence of change in the distribution of bases from the inoculum only in vaccinees, which could be indicative of mutations generated as a consequence of vaccination. All the sequences from animals R01093 and BB204 harboured a thymine at that position while a mixed population of thymine and cytosine was present in the inoculum. Since at least one of the inoculum genomes transmitted to these animals harboured a thymine at this position the most likely scenario is that this mutation was transmitted rather than generated *de novo* in independent animals as a result of vaccination. However, it is striking that in these 2 animals the entire viral population harboured a thymine at week 2 post-challenge and this residue persisted in the entire viral population at week 4 post-challenge.

Importantly the majority of the sites displaying a statistically significant difference in Shannon entropy between vaccinees and controls were also confirmed independently using the Bayesian model, thus the differential diversity observed at those positions was not the result of purely random events but very likely it was a predisposed outcome of the immunization protocol.

Finally, we estimated the selection pressures acting upon the *env* gene by calculating the global *dN/dS* ratio. The global *dN/dS* ratio for the complete data set was 0.56 consistent with the envelope gene being under purifying selection. We identified 2 codons under positive selection (164 and 829) and 8 codons under negative selection (46, 131, 251, 253, 438, 486, 588, 697) most of which were identified in a previous study where animals were challenged with the same inoculum [32]. We found no differences in the global *dN/dS* between controls and vaccinees (0.53 and 0.62 respectively).

## Discussion

NAbs have been shown to shape the intra-host evolution of the HIV-1 envelope [50]–[57] but little is known regarding their impact on early diversification events of breakthrough viral variants upon transmission. The analysis of the HIV-1 viral founding population of individuals enrolled in the STEP trial revealed that even thought the immunization regime had no clinical efficacy, CTL responses left a genetic imprint in the viral regions targeted by the vaccine [17]. Most importantly, analysis of the breakthrough viral population of the RV144 trial found 2 signature sites associated with vaccine-induced immune responses [19]. Similarly, studies performed in rhesus macaques infected with SIV showed that different mutational patterns developed in animals subject to immunization regimes in comparison with control animals [15], [16]. Here, we presented data showing that pre-existing envelope only targeted immunity left an imprint, although modest, in the early viral population evidenced by the fact that viruses infecting immunized animals harboured genomes that encoded envelopes that differed more from the vaccine inserts than non-immunized controls. This is to our knowledge the first experimental study showing the impact of immune responses on viral genomes leading to infection in a highly controlled setting provided by: 1) rhesus macaques infected with chimeric SIV/HIV virus as a challenge where the HIV-1 Env glycoprotein can be studied in an animal model and the time of infection is known as well as the viral population to which animals are exposed to; and 2) the use of Env-based antigens in isolation without any other confounding factors (antigens).

We first quantified the viral founder variants and we observed that vaccination did not affect the transmission bottleneck. This is not surprising since a single high dose challenge was used which lead to the transmission of multiple viral variants in all the animals. Although a lower number of genomes seemed to have been transmitted to animals of group 1 (median of 4 against a median of 6 in controls), this difference did not reach statistical significance probably due to the low number of animals available for retrospective analysis. Unfortunately no samples were available from animals belonging to group 2 that had a better vaccine-induced protection.

Global measures of intrahost variation such as mean pairwise distance, proportion of phylogenetic informative mutations or measures of evolutionary forces acting upon *env* failed to identify differences between vaccinated and control animals, which would suggest that this vaccination protocol did not affect the mutational dynamics of *env*. However, we found that *env* underwent different patters of diversification in controls and vaccinees: we found that although viruses infecting vaccinees were in general constrained to diversify away from the consensus sequence of the inoculum they tended to encode for Env proteins that in specific regions differed from the vaccine inserts, corresponding with the presence of binding antibodies towards those regions. Importantly, several sites-of-interest were also identified in the same regions. However, given the limitation on animal numbers inherent to non-human primate studies, these results should be interpreted with caution and within the context of this particular immunization protocol.

Similar to the scenarios proposed by Rolland et al. [17] for CTL-based vaccines in the presence of pre-existing immune responses generated through Env-based vaccination strategies, incoming viruses could take two different but not mutually exclusive routes upon transmission: i) viruses that are similar to the vaccine are filtered out by either neutralization or other antibody-mediated effector functions; or ii) viruses are forced to diversify away from the vaccine. The fact that we found no clustering of *env* sequences according to vaccination status and that the divergence in some of the regions of the viral *envs* encoded by vaccinees was higher in vaccinees than in controls point to the diversification away of the founder viruses from the vaccine “strain” as the mechanism ruling genetic variation in this animal model. We acknowledge that due to our acute post-infection period in which samples were available for sequence analysis (2 and 4 weeks post-challenge) it is not possible for a mutation that confers higher fitness to arise and replace the whole viral population [45]. Thus we attribute to this the cause of why the differences in divergence from the vaccine inserts between vaccinees and controls were modest and in some comparisons, they did not reach statistical significance. If we had used a more neutralisation resistant SHIV strain (which are inherently more pathogenic), we may have overwhelmed the modest vaccine effect completely and possibly have lost this observation. The analysis of later samples would have been beneficial but these were unfortunately not available. On the other hand the use of neutralisation sensitive SHIV model has the limitation of short periods of viremia, which only allows the study of early diversification events since all animals will rapidly clear the infection.

The poor clinical efficacy of this trial could be attributed in part to the stringent challenge protocol that the animals were subjected to: a single high intrarectal dose. Here we showed that all the animals were infected by multiple viral variants, a scenario that is unlikely to reflect natural HIV-1 infection. It could be argued that a better outcome could have been obtained if animals were infected with a single viral genome. We have previously shown that in terms of transmitted genotypes, a single challenge with an intermediate challenge dose better mimics the early diversification events seen in natural HIV-1 infection [32]. It would then seem sensible to re-evaluate the non-human primate challenge models to avoid “missing” potential vaccine candidates. Importantly, in light of recent findings, sieve analysis should now be considered critical part of the evaluation of immune correlates of protection and taken into account when powering pre-clinical vaccine trials in non-human primates.

In conclusion, we showed that an immunization protocol based only on Env antigens left an impact, although modest, on the viral population detected early in infection despite having little clinical efficacy. This is surprising considering the relatively small number of animals involved in the trial and the high stringency of the challenge protocol. Our results indicate that the analysis of the early viral population should be a critical component of the evaluation of efficacy of pre-clinical vaccine trials in non-human primates.

## Supporting Information

Table S1 (DOC)
