# Supplementary material for: Genetic Imprint of Vaccination on Simian/Human Immunodeficiency Virus Type 1 Transmitted Viral Genomes in Rhesus Macaques
Source: PLoS One. 2013 Aug 14;8(8):e70814. doi: 10.1371/journal.pone.0070814 (PMC3743870; doi:10.1371/journal.pone.0070814)
Supplement: Table S1 — (DOC) [file pone.0070814.s001.doc]

**Supplemental material**

**Table S1. Summary of the sequences analyzed.**

| **Animal ID** | **N^o^ sequences** | **N^o^ deletions** | **N^o^ insertions** | **N^o^ stop codons** | **N^o^ hypermutations** | **% diversity** |
| --- | --- | --- | --- | --- | --- | --- |
| **Inoculum** | 63 | 3 | 1 | 1 | 0 | 0.31 |
| **Group 1** |  |  |  |  |  |  |
| R00040 |  |  |  |  |  |  |
| Week 2 | 27 | 5 | 0 | 17 | 3 | 0.07 |
| Week 4 | 6 | 1 | 0 | 0 | 0 | 0.63 |
| R01093 |  |  |  |  |  |  |
| Week 2 | 24 | 7 | 0 | 18 | 0 | 0.2 |
| Week 4 | 33 | 6 | 0 | 0 | 0 | 0.16 |
| R99008 |  |  |  |  |  |  |
| Week 2 | 9 | 3 | 1 | 1 | 1 | 0.27 |
| Week 4 | 10 | 3 | 0 | 0 | 0 | 0.43 |
| **Group 3** |  |  |  |  |  |  |
| R00057 |  |  |  |  |  |  |
| Week 2 | 34 | 8 | 0 | 4 | 1 | 0.24 |
| Week 4 | 10 | 0 | 0 | 0 | 0 | 0.09 |
| BB204 |  |  |  |  |  |  |
| Week 2 | 25 | 8 | 0 | 12 | 6 | 0.15 |
| Week 4 | 8 | 0 | 0 | 8 | 0 | 0.13 |
| 8758 |  |  |  |  |  |  |
| Week 2 | 40 | 13 | 0 | 4 | 2 | 0.27 |
| Week 4 | 17 | 3 | 0 | 0 | 0 | 0.29 |
| **Group 4** |  |  |  |  |  |  |
| R00056 |  |  |  |  |  |  |
| Week 2 | 29 | 1 | 2 | 5 | 3 | 0.08 |
| Week 4 | 19 | 1 | 0 | 0 | 0 | 0.13 |
| R99004 |  |  |  |  |  |  |
| Week 2 | 19 | 4 | 1 | 1 | 0 | 0.1 |
| Week 4 | 41 | 3 | 0 | 3 | 2 | 0.28 |
| Ri102 |  |  |  |  |  |  |
| Week 2 | 27 | 1 | 0 | 0 | 0 | 0.25 |
| Week 4 | 17 | 0 | 0 | 0 | 0 | 0.22 |
| Ri112 |  |  |  |  |  |  |
| Week 2 | 34 | 2 | 1 | 3 | 2 | 0.15 |
| Week 4 | 20 | 2 | 0 | 0 | 0 | 0.17 |
